# Autophagy and Lysosomal Functionality in CMT2B Fibroblasts Carrying the RAB7^K126R^ Mutation

**DOI:** 10.3390/cells11030496

**Published:** 2022-01-31

**Authors:** Roberta Romano, Victoria Stefania Del Fiore, Paola Saveri, Ilaria Elena Palamà, Chiara Pisciotta, Davide Pareyson, Cecilia Bucci, Flora Guerra

**Affiliations:** 1 Department of Biological and Environmental Sciences and Technologies, University of Salento, Via Provinciale Lecce-Monteroni n. 165, 73100 Lecce, Italy; roberta.romano@unisalento.it (R.R.); victoriastefania.delfiore@unisalento.it (V.S.D.F.); 2 Department of Clinical Neurosciences, Fondazione IRCCS Istituto Neurologico Carlo Besta, 20133 Milan, Italy; paola.saveri@istituto-besta.it (P.S.); chiara.pisciotta@istituto-besta.it (C.P.); davide.pareyson@istituto-besta.it (D.P.); 3 Nanotechnology Institute, CNR-NANOTEC, Via Monteroni, 73100 Lecce, Italy; ilaria.palama@nanotec.cnr.it

**Keywords:** Charcot-Marie-Tooth, RAB7A, autophagy, lysosome, lipophagy, lipid droplets

## Abstract

Charcot-Marie-Tooth type 2B (CMT2B) disease is a dominant axonal peripheral neuropathy caused by five mutations in the *RAB7A* gene. Autophagy and late endocytic trafficking were already characterized in CMT2B. Indeed, impairment of autophagy and an increase in lysosomal degradative activity were found in cells expressing the mutant proteins. Recently, we described a novel *RAB7* mutation associated with predominantly motor CMT2 and impaired EGFR trafficking. With the aim to analyze the autophagy process and lysosomal activity in CMT2B fibroblasts carrying the p.K126R RAB7 novel mutation and to investigate further the causes of the different phenotype, we have performed Western blot, immunofluorescence and cytometric analyses monitoring autophagic markers and endocytic proteins. Moreover, we investigated lipophagy by analyzing accumulation of lipid droplets and their co-localization with endolysosomal degradative compartments. We found that cells expressing the RAB7^K126R^ mutant protein were characterized by impairment of autophagy and lipophagy processes and by a moderate increase in lysosomal activity compared to the previously studied cells carrying the RAB7^V162M^ mutation. Thus, we concluded that EGFR trafficking alterations and a moderate increase in lysosomal activity with concomitant impairment of autophagy could induce the specific predominantly motor phenotype observed in K126R patients.

## 1. Introduction

Charcot-Marie-Tooth (CMT) is a rare inherited disorder affecting the peripheral nervous system. CMT type 2B (CMT2B) is an axonal form of the disease characterized by prominent sensory loss, progressive distal weakness leading to atrophy, reduced tendon reflexes, normal or near-normal nerve conduction velocities but also foot deformities, ulcers and infections leading to toe and foot amputations [1,2,3]. Because of the frequency of amputations, it is also classified as an ulcero-mutilating neuropathy [1,2,3]. At present, CMT is classified as an incurable pathology, and physical therapy, occupational therapy and orthopedic surgery and/or devices are the only treatments available for patients.

Autosomal dominant inheritance of the disease is ascribed to five missense mutations (p.L129F, p.K157N, p.N161T/I, and p.V162M) in the *RAB7A* gene, which encodes a guanosine triphosphatase (GTPase) of the RAB (Ras-related in brain) family [4,5,6,7]. RAB7A, hereafter referred to as RAB7, is a well-characterized ubiquitous GTPase with multiple important roles in cellular physiology, in particular in the regulation of late endocytic traffic from endosomes to lysosomes and of lysosomal biogenesis [8,9,10]. Consequently, RAB7 is also important for other processes in which the late endocytic route is involved, such as phagosome and autophagosome maturation, interaction and fusion between phagosomes and autophagosomes with lysosomes, and thus phagolysosome and autolysosome biogenesis [11,12,13]. RAB7 also intervenes in specialized autophagy. Indeed, it regulates the shape of the nascent autophagosome isolation membrane around damaged mitochondria during mitophagy and the breakdown of lipid droplets (LDs) during lipophagy [14,15]. Moreover, apoptosis, membrane channel trafficking, retromer recruitment and functioning are other processes in which RAB7 is involved [8,16,17,18]. Notably, RAB7 has been demonstrated to play specific roles in neurons where it regulates neurotrophin trafficking and signaling, neurite outgrowth and neuronal migration during development [19,20,21].

Recently, we have characterized clinically and genetically a novel *RAB7A* mutation (c. 377A > G, p.K126R) predicted to be deleterious. We performed structural and functional analysis of the mutant protein comparing it to the p.V162M classical CMT2B mutant protein [22]. We initially found a substantial difference in the phenotypic manifestation of the disease. Indeed, CMT2B, in general, is characterized by mild-to-absent motor deficits [3,23] while, in the patient harboring the p.K126R mutation, electrophysiology demonstrated an axonal neuropathy with predominant motor involvement, early-onset walking difficulties, progressive distal muscle wasting and weakness, particularly in lower limbs, and only mild sensory signs [22].

We previously analyzed the biochemical properties of the RAB7^K126R^ mutant in terms of GDP and GTP dissociation rate constants (Koff) and demonstrated that this mutant displays higher nucleotide Koff (higher for GDP than for GTP) and impaired GTP hydrolysis per binding event, similarly to previously analyzed CMT2B-causing mutant proteins [24,25,26]. Moreover, we found that the RAB7^K126R^ mutant has a very strong inhibitory effect on neurite outgrowth similarly to the other CMT2B-causing RAB7 mutants [27,28] with obvious implications on axonal regeneration [29]. Indeed, considering that the onset of CMT2B occurs in the second or third decade of life, aging of these patients could lessen the effects of factors that counteract the detrimental action of the RAB7 mutant proteins, and, finally, could determine the reduction in regeneration capabilities contributing to the progressive axonal loss. Another feature of RAB7^K126R^ is its ability to interact more strongly with peripherin compared to RAB7^wt^, again behaving similarly to the other CMT2B causing RAB7 mutant proteins [30]. To this purpose, it is important to note that neuronal morphology, maturation and differentiation, but also axonal regeneration are processes in which peripherin is involved and is very important [31,32]. Moreover, we have previously shown that RAB7 influences peripherin assembly and alterations of this interaction detected in CMT2B could be among the causes of axonal regeneration impairment [30]. Although for most of the phenotypes studied we found no significant distinctions among those induced by the K126R and those induced by the other CMT2B-causing *RAB7* mutations, we were able to find a difference. Indeed, in skin patient’s fibroblasts carrying the K126R mutation, we found a strong accumulation of Epidermal Growth Factor Receptor (EGFR) caused by inhibition of EGFR degradation, in contrast to what has been observed for fibroblasts carrying the V162M classical CMT2B mutation [22,33]. Furthermore, expression of the classical CMT2B-causing RAB7 mutant proteins in HeLa cells does not inhibit EGFR degradation nor induces its accumulation, in contrast to what happens if the K126R mutant protein is expressed [22,24,25]. In addition, in patient fibroblasts with the K126R mutation, EGFR remains in the early endosomes and impairment of EGFR trafficking to late endosomes and lysosomes determines inhibition of EGFR degradation with consequent accumulation. Thus, there is an interesting molecular difference between fibroblasts harboring the K126R mutation and fibroblasts harboring the other CMT2B-causing mutations although how this affects the motor phenotype detected in the patient remains obscure.

Late endocytic trafficking and autophagy are closely related processes, and RAB7, regulating maturation of late endosomes and lysosomal biogenesis, is also implicated in autophagosome maturation and fusion with lysosomes [8]. We have already characterized autophagy in CMT2B and we found that RAB7 mutants determine the impairment of the autophagic flux [34]. Moreover, it is known that LD breakdown is regulated by RAB7 through CD63 and LAMP-1 positive degradative compartments [15] and we have previously demonstrated dysfunctional lipophagy in CMT2B with accumulation of bigger LDs compared to healthy cells [35].

In light of these previous pieces of knowledge, we decided to further investigate the CMT2B-causing RAB7^K126R^ mutant protein by looking at the effects of its expression on autophagy, lipophagy, endocytosis and lysosomal functionality, with the aim to deepen the knowledge on it.

## 2. Materials and Methods

### 2.1. Antibodies and Reagents

Primary antibodies used in this study were the following: anti-SQSTM1/p62 (1:1000; 610833) from BD Biosciences (Franklin Lakes, NJ, USA), anti-MAPK1/ERK2 (1:1000; sc-154) from Santa Cruz Biotechnology (Dallas, TX, USA), anti-LC3B (1:500 for WB, 0231–1000) from Nanotools (Teningen, Germany), anti-LC3B (1:50 for IF, M152-3) from MBL (Woburn, MA, USA), anti-LAMP1 (1:250, H4A3), deposited to the Developmental Studies Hybridoma Bank (University of Iowa, Iowa City, IA, USA) by J.T. August and J.E.K. Hildreth, anti-CD63 (1:50; Santa Cruz Biotechnology, sc-15363), anti-Cathepsin D from Santa Cruz Biotechnology (1:500, sc-6486), anti-RAB5 (1:200, Santa Cruz Biotechnology, sc-309), anti-RAB4 (1:200, Santa Cruz Biotechnology, sc-312), anti-RAB9 (1:1000, ab3810) from Abcam (Cambridge, UK), Anti-CI-MPR (1:1000, Abcam, ab32815), anti-TGN46 (1:500, AHP500) from Bio-Rad (Hercules, CA, USA), anti-TSG101 (1:500, Abcam, ab125011), anti-EAP30 (1:200, Santa Cruz Biotechnology, sc-390748). The HRP-conjugated secondary antibodies used for Western blot analysis were from Bio-Rad (Hercules, CA, USA) (anti-mouse #1706516; anti-rabbit #1706515) or from Santa Cruz Biotechnology (anti-sheep #16047). The secondary antibodies used in immunofluorescence experiments were the following: anti-mouse Alexa Fluor 488-conjugated (A21202), anti-rabbit Alexa Fluor 488-conjugated (A21206), anti-mouse Alexa Fluor 555-conjugated (A31570), anti-rabbit Alexa Fluor 555-conjugated (A31572), all from Life Technologies (Carlsbad, CA, USA). 

Bafilomycin A1 (Santa Cruz Biotechnologies, sc-201550) was dissolved in DMSO. EBSS (E2888) (Earle’s Balanced Salt Solution) and Hanks’ medium (H9269) were used as starvation media and were purchased from Sigma-Aldrich (St. Louis, MO, USA). 0.5% Oil Red-O/isopropyl alcohol solution was obtained from Sigma-Aldrich (O1391) and BODIPY 493/503 from ThermoFisher (D3922) (Waltham, MS, USA).

### 2.2. Cell Lines

HeLa cells (ATCC CCL-2; human cervix adenocarcinoma) were grown in Dulbecco’s modified Eagle medium (DMEM) containing 10% FBS (Fetal Bovine Serum), 2 mM L-glutamine, 100 U/mL penicillin and 10 mg/mL streptomycin.

Dermal fibroblasts derived from an age- and sex-matched healthy control, a patient carrying the RAB7A^V162M^ mutation (patient III.7 from the first Italian family affected by CMT2B) and from another patient with the RAB7A^K126R^ mutation (patient III.1) were isolated as previously described [3,22,33]. These cells were grown in DMEM supplemented with 15% FBS, 2 mM L-glutamine, 100 U/mL penicillin and 10 mg/mL streptomycin. Cell culture reagents were obtained from Gibco (Amarillo, TX, USA).

All cell lines were cultivated in 5% CO_2_ incubator at 37 °C and were confirmed to be contamination-free.

Informed consent was obtained in compliance with the Helsinki Declaration for all procedures from all study participants. All samples were anonymously encoded to protect patient confidentiality.

### 2.3. Plasmids and Transfection

Plasmids coding for HA-tagged RAB7A^wt^ and the CMT2B-causing mutant proteins RAB7A^V162M^ and RAB7A^K126R^ were previously described [22,24]. In transfection experiments, we used the empty vector pcDNA3_2xHA as control. In co-transfection experiment (see Section 2.8) we used pBABE-puro mCherry-EGFP-LC3B.

Transfection was performed with Metafectene Pro (Biontex, Martinsried, Germany) according to the manufacturer’s instruction. Cells were fixed 48 h after transfection for immunofluorescence experiments.

### 2.4. Western Blot Analysis

Cells were lysed in Laemmli buffer [(100 mM Tris-HCl, pH 6.8, 4% (*w*/*v*) SDS, 0.2% (*w*/*v*) bromophenol blue, 20% glycerol and 200 mM DTT (dithiothreitol)] and then processed for Western blot analysis, as previously described [33,36]. Briefly, after SDS-PAGE, proteins were transferred on PVDF membrane (Merck-Millipore, Burlington, MS, USA) which was then blocked with 5% milk in PBS for 30 min and then incubated with the appropriate primary antibodies (over-night at 4 °C) and secondary HRP-conjugated antibodies (1 h at room temperature), all prepared in 1% milk in PBS. Chemiluminescence reaction occurred using Clarity or Clarity Max (Biorad, Hercules, CA, USA) and the signal was captured by ChemiDoc MP Imaging Systems (Biorad). Densitometric analysis was performed using Image Lab software (Biorad).

### 2.5. Oil-Red O Staining

For lipid droplets staining, cells were treated as previously described [35]. Briefly, cells were seeded on 11 mm round glass and when they reached 80% confluency, they were washed with PBS and then fixed with 3% paraformaldehyde in PBS for 20 min at room temperature. After fixation, 60% isopropanol was used to wash cells for 5 min and then left to dry. Cells were treated with a 0.5% Oil Red-O/isopropyl alcohol solution for 20 min and then washed abundantly with distilled water. Nuclei were stained with 1 μM diamidino-2-phenylindole (DAPI) for 5 min. Lipid droplets were visualized using EVOS FL Auto Cell Imaging System (ThermoFisher, Waltham, MS, USA) with PlanApo N 60×/1.42 oil (Olympus, Shinjuku, Tokyo, Japan).

### 2.6. Immunofluorescence, Confocal Microscopy and Live Imaging

Cells were grown on 11 mm round glass. Permeabilization was performed using 0.25% saponin in 80 mM PIPES (pH 6.8), 5 mM EGTA and 1 mM MgCl_2_ for 5 min at room temperature and then cells were fixed with 3% paraformaldehyde in PBS for 20 min at room temperature. The incubation with primary and secondary antibodies occurred at room temperature for 20 min. All antibodies were diluted in 0.1% saponin in PBS. Nuclei were stained with DAPI for 5 min. After several washes, coverslips were mounted with Mowiol and images were captured with Zeiss LSM700 confocal laser scanning microscope (Zeiss, Oberkochen, Germany) equipped with a 63×/1.40 NA oil immersion objective.

For the BODIPY staining, cells were washed with PBS and incubated with 2 µM BODIPY 493/503 for 15 min at 37 °C. After that, cells were washed twice with PBS and then fixed, permeabilized and stained with the appropriate antibodies as previously described.

For live microscopy, cells were seeded into microscopy chambers (8-well μ-slide, Ibidi GmBh, Martinsried, Germany) and, after 24 h, incubated with 1 μM LysoTracker Red DND-26 for 30 min at 37 °C, as previously described [37,38]. This dye was from ThermoFisher Scientific (Carlsbad, CA, USA) and is characterized by amine groups, which are partially protonated at neutral pH and fully protonated at acidic pH. In particular, LysoTracker DND-26 fluorescence is somewhat pH-independent.

Intensity of fluorescence was determined by ImageJ software (Version 1.5Oi, Bethesda, MD, USA) and it was calculated as intensity/cell normalizing on the area of each single cell [33]. Measures were obtained by analyzing at least 20 cells/sample in at least three independent experiments.

### 2.7. DQBSA (Self-Quenched BODIPY Dye Conjugates of Bovine Serum Albumin) Assay

Cells were seeded on 11 mm round glass coverslips and incubated in the presence of Red DQ-BSA (50 μg/mL) (ThermoFisher Scientific, Carlsbad, CA, USA) for 24 h at 37 °C with full medium in a humidified atmosphere of 5% CO_2_. Subsequently, cells were fixed with 3% paraformaldehyde in PBS for 20 min at room temperature, incubated with DAPI dye (1 μg/mL) and analyzed with LSM 700 confocal microscope (Zeiss). Emission intervals: Red DQBSA = 560–615 nm (λ_ex_ = 555 nm). Images were taken with a Plan-Apochromat 63×/1.40 oil-immersion objective DIC M27 and the pinhole aperture was set to 1 Airy unit. Images were acquired using ZEN Black Edition 2011 acquisition software (Zeiss, Germany).

Intensity of fluorescence was determined by ImageJ software (Version 1.5Oi, Bethesda, MD, USA). Intensity of fluorescence was calculated as intensity/cell normalizing on the area of each single cell. Measures were obtained by analyzing at least 20 cells/sample for at least three different experiments.

### 2.8. Flow Cytometry

HeLa cells, 48 h after co-transfection, were treated with full medium and starvation medium for 4 h. After, they were harvested and resuspended in PBS1X for flow cytometer analysis using a CytoFLEX S (Beckman Coulter, Brea, CA, USA) equipped with 488 nm and 561 nm laser. The ratio mCherry and EGFP signal was created as channel in the CytExpert software (version 2.4.0.28, Beckman Coulter, Brea, CA, USA) in order to determinate the autophagic flux [39]. In particular, the flux in each cell was calculated creating a customized parameter in CytExpert software in which the quotient of the mCherry and EGFP fluorescence channels were calculated with the followed equation:

mCherry: EGFP ratio = *F_mCherry_/F_EGFP_* (1)

where mCherry:EGFP ratio represents autophagic flux, F_mCherry_ represents the fluorescence of mCherry and F_EGFP_ represents the fluorescence of EGFP.

### 2.9. Statistical Analysis

Intensity of fluorescence was determined by ImageJ software (Version 1.5Oi, Bethesda, MD, USA). Measures were obtained by analyzing at least 50 cells/sample and at least 20 cells/sample for at least three different experiments, for HeLa cells and fibroblasts respectively. Fluorescence intensity was evaluated quantifying Correct Total Cell Fluorescence (CTCF), while number and size of vesicles through Analyze Particle’ tool of ImageJ software [40]. Colocalization rate was determined by Zen 2011 software (Carl Zeiss, Oberkochen, Germany) as the weighted colocalization coefficient of RAB7 and LC3B and LDs and CD63/LAMP-1. All experiments were repeated at least three times and the error bars represent the standard error of the mean (S.E.M.). All statistical *t*-test analysis performed on data obtained from treated cells were performed between the same conditions (FM, ST and BAF) selecting CTR fibroblasts or HeLa cells transfected with RAB7^wt^ as the referring samples (* *p* < 0.05, ** *p* < 0.01 and *** *p* < 0.001).

## 3. Results

### 3.1. Altered Autophagic Flux in CMT2B Fibroblasts

To investigate the role of CMT2B-causing RAB7^K126R^ mutant protein on autophagy, we monitored LC3B-II by Western blotting analysis on healthy individual and CMT2B patient-derived fibroblasts, the latter harboring K126R or V162M mutations. Notably, fibroblasts from patients with the RAB7^V162M^ mutation were used as a positive control because we previously demonstrated impairment of the autophagic process in these cells [34].

**Figure 1:**
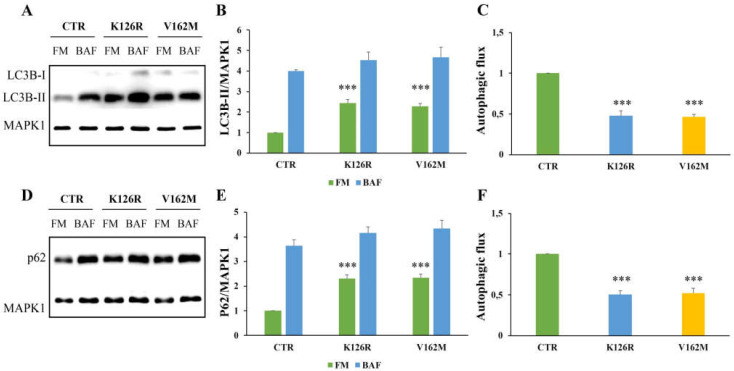
Analysis of autophagy in CMT2B fibroblasts by Western blotting. Control fibroblasts (CTR) and cells carrying K126R and V162M mutations were incubated with full medium (FM) and with 400 nM of Bafilomycin A_1_ (BAF) for 3 h. (**A**,**D**) Western blotting analysis was performed on cellular lysates from healthy control fibroblasts and fibroblasts carrying RAB7^K126R^ and RAB7V^162M^ mutations using specific antibodies against LC3B and p62. MAPK1 was used as loading control. Densitometric analysis of immunoblot shown in (**B**) and (**E**) was performed using Image Lab software (BIO-RAD). The autophagic flux was calculated as the ratio of normalized LC3B-II (**C**) and p62 (**F**) between BAF and FM of the same sample. Values are the mean ± standard error (SE) of three different and independent experiments. *** *p* < 0.0001.

The cells were incubated in parallel with full medium and with bafilomycin A1, which inhibits the vacuolar type H^+^-translocating ATPase (V-ATPase) and is used to prevent lysosomal turnover of the autophagosome content [41]. As shown in Figure 1A,B, LC3B-II was present in higher amounts in both CMT2B fibroblasts grown in full medium. However, quantification of autophagic flux by measuring the ratio of LC3B-II expression levels between bafilomycin A_1_ and full medium of the same sample revealed a strong inhibition in CMT2B cells carrying V162M or K126R mutations compared to control cells (Figure 1C).

In addition, we analyzed the autophagic flux monitoring SQSTM1/p62 (referred only as p62) after 24 h of treatment with bafilomycin A_1_ [41]. Also, in this case, we found a significant increase in p62 protein in CMT2B fibroblasts grown in full medium and a strong reduction in the autophagic flux, calculated by ratio of p62 expression levels between bafilomycin A_1_ and full medium of the same sample, and compared to the healthy control cells (Figure 1D–F).

**Figure 2:**
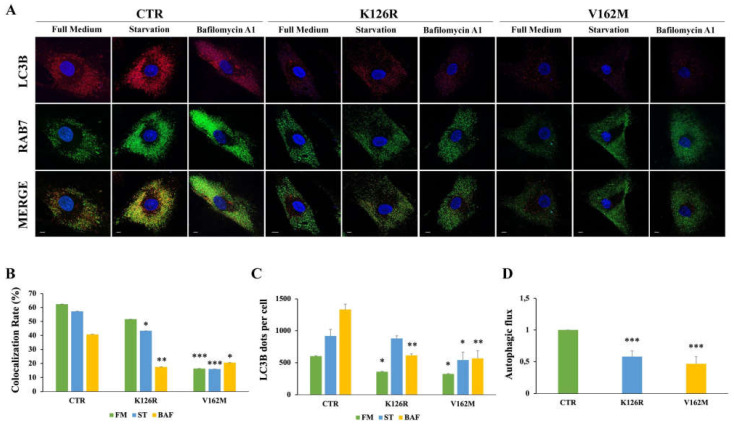
Analysis of autophagy in CMT2B fibroblasts by immunofluorescence. Control fibroblasts (CTR) and fibroblasts carrying K126R and V162M mutations were incubated with full medium (FM) or starvation medium (ST) for 30 min or incubated with 400 nM of Bafilomycin A_1_ (BAF) for 3 h. (**A**) LC3B dots for cells and colocalization with RAB7 was evaluated by immunofluorescence using antibodies against LC3B (red) and RAB7 (green). Nuclei were stained with DAPI (blue). (**B**) Colocalization rate was analyzed for each condition (FM; ST and BAF) of the same sample. (**C**) The number of autophagosomes (scored as LC3B dots) per cell was evaluated for each sample. (**D**) The autophagic flux was calculated as the ratio of LC3B dots between BAF and FM of the same sample. Values are the mean ± SE of three different and independent experiments. * *p* < 0.05; ** *p* < 0.01; *** *p* < 0.0001. Scale bar = 10 µm.

In order to further validate these data, we performed immunofluorescence analysis on healthy and CMT2B fibroblasts incubated with full medium, with starvation medium to induce the autophagic process, and in the presence of bafilomycin A1 (Figure 2A). We analyzed the colocalization rate of RAB7 protein with LC3B in the three conditions. We found a reduction in colocalization in CMT2B cells compared to control cells, but interestingly we have observed less reduction in K126R (~10%) in full medium and in starvation medium compared to V162M (~70%) in the same conditions. Notably, when we treated these cells with bafilomycin A1, the rate of RAB7/LC3B colocalization was similarly reduced in both cell lines (of about 50%) compared to healthy fibroblasts (Figure 2B).

These results indicate that in CMT2B cells RAB7 is less recruited on autophagic compartments compared to control cells, indicating the influence of *RAB7* mutations in the autophagic process.

The main mechanistic factor that regulates the amplitude of the autophagic activity is represented by the number of autophagosomes. Using confocal microscopy analysis, we observed a significant reduction in LC3B positive vesicles in CMT2B fibroblasts carrying the RAB7^V162M^ mutation in each of the conditions tested, confirming previously obtained data [34]. We found a significant reduction in LC3B dots per cell also in CMT2B patient cells carrying the RAB7^K126R^ mutation both in full medium and upon bafilomycin A1 treatment, but, interestingly, when we starved these cells, autophagic vesicles were not reduced (Figure 2C).

It is important to note that a comparison of immunofluorescence and immunoblotting data showed that, while fewer LC3 vesicles were detected by immunofluorescence in CMT2B fibroblasts grown in full medium (Figure 2A,C), immunoblotting in the same conditions revealed an increase in LC3B-II (Figure 1A,B). The differences detected could be due to the different antibodies employed but also to the detection limits of fluorescence microscopy. Indeed, in the case of immunofluorescence, only signals that exceed a certain threshold will be detected. Thus, for instance, autophagic vesicles carrying few LC3B molecules will not be visible. Therefore, this result could suggest accumulation of autophagic vesicles with a low number of LC3B molecules per organelle. Despite these differences, we observed a significant reduction in the autophagic flux, both by immunofluorescence and by Western blot analysis, in CMT2B fibroblasts harboring the RAB7^K126R^ mutation indicating that impairment of autophagy is a common feature of CMT2B (Figure 2D).

### 3.2. Transfection of CMT2B-Causing RAB7^K126R^ Mutants Induces Impairment of the Autophagic Process

**Figure 3:**
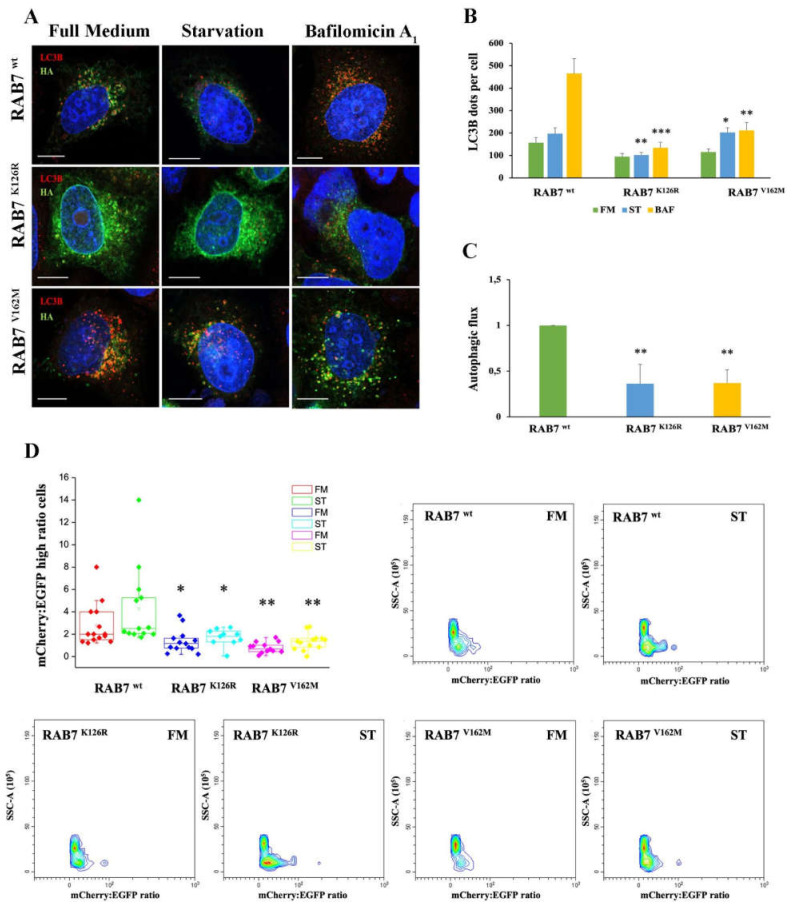
Analysis of autophagy in HeLa cells expressing RAB7^K126R^ and RAB7^V162M^ mutant proteins. (**A**) HeLa cells transfected for 48h with plasmids encoding HA-tagged RAB7^wt^ and CMT2B-causing RAB7 mutants (RAB7^K126R^ and ^V162M^) were incubated in full medium (FM) or starvation medium (ST) for 30 min or in the presence of 400 nM bafilomycin A_1_ (BAF) for 3 h. LC3B and HA-RAB7 proteins were identify using antibodies against LC3B (red) and HA (green), respectively. Nuclei were stained with DAPI (blue). (**B**) The number of LC3B-positive dots per cell was evaluated for each sample. (**C**) The autophagic flux was calculated as the ratio of LC3B dots between BAF and FM of the same sample. Scale bar = 10 µm. (**D**) HeLa cells co-transfected for 48 h with EGFP-mCherry-LC3B and with plasmids encoding HA-tagged RAB7^wt^ and CMT2B-causing RAB7 mutants (RAB7 ^K126R^ and ^V162M^) were incubated with full medium (FM) and starvation medium (ST) for 4 h. Scatter and singlet gates were used to eliminate debris, dead cells and mitotic cells. For each experiment, voltages and gain on EGFP and mCherry detectors are set empirically on negative and positive controls to allow the best plot fit on the mCherry and EGFP ratio histogram. Flow cytometry pseudo color plots of mCherry:EGFP ratio are reported. Statistical *t*-test analysis was performed between the same conditions (FM, ST and BAF) selecting RAB7^wt^ as the referring sample. All statistical comparisons are from the sample indicated with asterisks and RAB7^wt^. Values are the mean ± SE of three different and independent experiments. * *p* < 0.05; ** *p* < 0.01; *** *p* < 0.0001.

To further investigate the role of RAB7^K126R^ mutation on autophagy and to confirm data obtained in patient’s fibroblasts, we expressed HA-tagged RAB7^wt^, RAB7^K126R^ and RAB7^V162M^ proteins in HeLa cells (Figure 3A). In these cells, through immunostaining and confocal microscopy, we analyzed LC3B positive vesicles after incubation with full medium, starvation medium and after treatment with bafilomycin A1. Notably, we observed that expression of CMT2B-causing RAB7^K126R^ mutant protein, similarly to the RAB7^V162M^ mutant, determined a reduction in the amount of autophagic vesicles in starved cells and upon bafilomycin A1 treatment compared to RAB7 wild-type transfected cells (Figure 3B). Moreover, confirming the data obtained in CMT2B fibroblasts, expression of the RAB7^K126R^ mutant protein in HeLa cells caused a significant and strong autophagic flux decrease similar to what happens upon expression of the RAB7^V162M^ mutant protein (Figure 3C).

Furthermore, we used tandem EGFP-mCherry-LCB3 to monitor autophagic vesicles maturation with the aim to understand in which step the CMT2B RAB7^K126R^ mutant protein inhibits autophagy (Figure 3D). Indeed, EGFP fluorescence is sensitive to the acid environment of autolysosomes compared to mCherry fluorescence. For this reason, a lower green signal is detected in cells with higher autophagic flux as a consequence of the autophagosome–lysosome fusion determining an increase in mCherry/EGFP ratio in the cells. This ratio was calculated using flow cytometry and used as a measurement of autophagic flux in each cell [39]. By this method, we found that CMT2B-causing RAB7^K126R^ mutant expressing cells were characterized by a reduction in autophagosome/lysosome fusion rate compared to RAB7 wild-type transfected cells and this decrease was comparable in cells expressing CMT2B-causing RAB7^V162M^ mutant (Figure 3D).

These results demonstrated that CMT2B RAB7^K126R^ mutant protein affects the autophagic process determining its impairment.

### 3.3. RAB7^K126R^ Mutant Protein Affects the Lipophagy Mechanism

**Figure 4:**
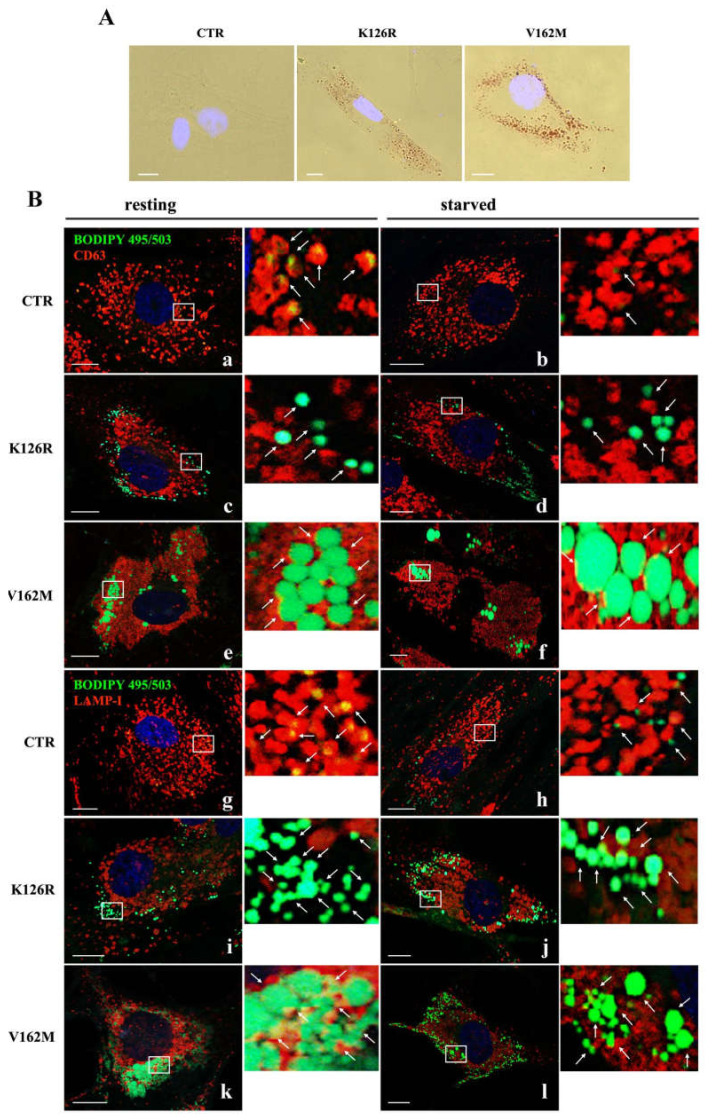
Lipid droplet (LD) accumulation in CMT2B fibroblasts. (**A**) Microscopic observations of the Oil Red-O dye-stained LDs from control (CTR) and CMT2B’s patient’s fibroblasts (K126R and V162M). (**B**) LD interaction with multivesicular bodies (MVBs) and lysosomes was investigated by immunofluorescence analysis using BODIPY 493/503 to label LDs (green) and anti-CD63 (red) and anti-LAMP-I (red) antibodies in cells incubated with full medium (resting condition) and starvation medium (starved condition). White arrows point to LDs. Nuclei were stained with DAPI (blue) White boxes indicated zoomed areas on the right. Scale bar = 10 µm.

In previous work, we demonstrated that expression of the RAB7^V162M^ mutant protein determines LD accumulation due to alterations in LD breakdown. In order to establish if RAB7^K126R^ is associated with the same phenotype, we decided to assess LD accumulation in CMT2B RAB7^K126R^ fibroblasts by a Red-O dye staining experiment [35]. We found that fibroblasts bearing the RAB7^K126R^ mutation also have more lipid accumulation in LDs compared to control cells where, with this method, LDs were not visible in most cells (Figure 4A). However, although RAB7^K126R^ mutant cells accumulated more LDs compared to healthy fibroblasts, accumulation and size of LDs were much less than in RAB7^V162M^ fibroblasts.

In order to confirm these data and to analyze localization and lipophagy in K126R fibroblasts, we grew the cells in full medium or incubated them in EBSS buffer for 24 h. Staining of LDs with BODIPY 493/503 and their recruitment in CD63-positive multivesicular bodies (MVBs) or in LAMP1-positive lysosomes were analyzed through confocal microscopy [15]. As expected, BODIPY-labeled LDs were present in control cells, uniformly distributed in the cytosol (Figure 4B, panels a, b, g and h). Much stronger staining in all CMT2B cells revealed many more LDs (Figure 4B). Nevertheless, in K126R cells, LD size was smaller than in CMT2B V162M cells, although we found a similar peripheral distribution (Figure 4B, panels c–f and i–l), confirming data obtained with Oil-Red-O staining (Figure 4A).

Moreover, although the difference in size between K126R and V162M persisted, in both cases, physical interactions with CD63-labeled MVBs in resting (full medium) and in starved conditions were very limited under both conditions compared to control cells (Figure 4B, panels c–f). Similarly, in control fibroblasts, LDs were surrounded by LAMP1-positive lysosomes in resting condition and after starvation (Figure 4B, panels g–h), while degradative LAMP-1 positive compartments were mainly not associated with LDs in the patient’s fibroblasts (K126R and V162M) (Figure 4B, panels i–l).

Quantitative analysis of LDs showed that CMT2B fibroblasts carrying RAB7^K126R^ mutation had a significant increase in the number of lipid droplets in resting and in starved conditions compared to control cells and similarly to CMT2B with the classical V162M mutation (Table 1). Interestingly, the evaluation of the average size of LDs showed that RAB7^K126R^ fibroblasts increased their size only during starvation, in contrast with RAB7^V162M^ cells in which LD size was greater both in resting and starving conditions. Finally, quantitative analysis of LD colocalization with CD63 and LAMP-1 positive endocytic compartments showed that in all conditions in CMT2B fibroblasts, LDs were less surrounded by multivesicular bodies (MVBs) and lysosomes indicating the occurrence of impairment of the lipophagy process (Table 1).

Altogether these results demonstrated that altered breakdown of LDs is also a feature of CMT2B RAB7^K126R^ mutant cells similarly to V162M fibroblast cells.

### 3.4. Lysosomal Activity Was Increased in Patient’s Fibroblasts Carrying the CMT2B-Causing RAB7^K126R^ Mutation

**Figure 5:**
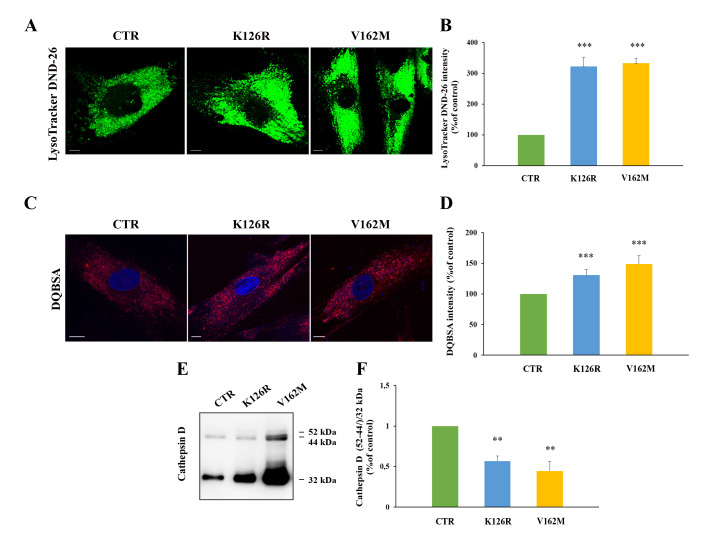
Alterations of late endocytic compartments in K126R mutated fibroblasts. (**A**) Cells were labeled with LysoTracker DND-26 (green) and (**B**) fluorescence intensity was quantified by ImageJ software. (**C**) Cells were incubated in the presence of DQ-Red BSA and (**D**) fluorescence intensity analyzed by ImageJ software. Nuclei were labeled with DAPI (blue). Scale bars = 10 μm. (**E**) Relative abundance of the three Cathepsin D forms was assessed by Western blotting and (**F**) quantified by densitometry normalizing against the 32 kDa mature form. Values are the mean ± SE of three different and independent experiments. ** *p* < 0.01; *** *p* < 0.0001.

In previous work, we analyzed the effect of RAB7^V162M^ mutant on endosomal trafficking and lysosomal functionality, and we found that this mutation determined higher lysosomal degradative activity [33]. With the aim to investigate if RAB7^K126R^ mutation induces the same effect on lysosomal activity, we analyzed in first instance lysosome abundances through live imaging assay using LysoTracker Green DND-26 staining on fibroblasts from CMT2B patients. As shown in Figure 5A, analysis of LysoTracker intensity revealed that K126R and V162M mutants were characterized by a comparable increase in lysosomal abundance with respect to fibroblasts from a healthy control (Figure 5B). 

In order to understand if K126R mutant was characterized by increased activity of late endocytic compartments, we decided to perform a DQ-Red BSA assay to detect lysosomal proteolytic activity (Figure 5C) [42]. This dye is strongly self-quenched by conjugation to BSA, but digestion of BSA determines its dequenching and the release of dye-labeled brightly fluorescent protein fragments. Furthermore, DQ-BSA is insensitive to pH from pH 3–11 allowing the direct detection of proteolytic activity in situations where the pH is unknown and cannot be controlled or where the pH is known to be low (e.g., endosomes and lysosomes). The quantification of DQ-BSA showed an increase in its intensity in K126R fibroblasts (30% ± 9.2%) compared to control fibroblasts and a decrease compared to V162M mutant (50% ± 13%), indicating that RAB7 K126R is accompanied by a slight increase in lysosomal activity compared to the classical CMT2B-causing RAB7^V162M^ mutant.

In order to confirm the alterations suggested by the DQBSA assay, we monitored Cathepsin D maturation in the three cell lines through Western blot analysis (Figure 5E).

Indeed, Cathepsin D is a lysosomal protease synthesized as preprocathepsin D precursor, converted into procathepsin D (52 kDa) in the endoplasmic reticulum, and further processed in the acidic milieu of late endosomes and lysosomes, into the 44-kDa form and finally into the 32-kDa mature form [43]. Accumulation of immature forms is used as marker of lysosomal impairment [33,37]. Maturation of Cathepsin D was evaluated through Western blotting analysis with an anti-Cathepsin-D antibody that recognizes not only the 32 kDa mature form but also the 52 kDa and 44-kDa immature forms. The amount of maturation was expressed as the ratio between immature forms (52/44 kDa) and mature form (32 kDa), as reported in Figure 5E.

The ratio between the two immature forms and the 32-kDa mature form was confirmed altered in CMT2B-causing RAB7^V162M^ expressing cells, as previously published [33], while an intermediate behavior between cells expressing RAB7^V162M^ and control cells was observed in cells expressing RAB7^K126R^ mutant protein, indicating that this mutation is able to increase the degradative activity, but not as much as the V162M mutation.

**Figure 6:**
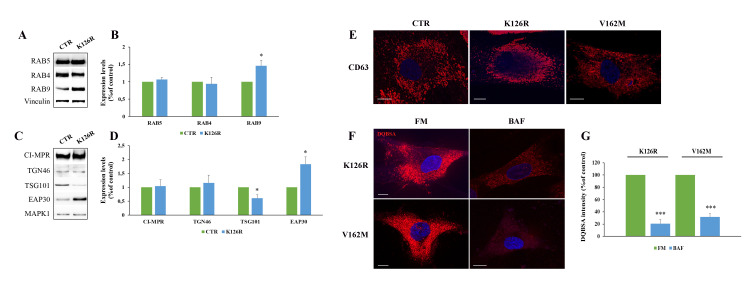
Analysis of endosomal markers in K126R mutated fibroblasts. (**A**,**C**) Lysates of control and CMT2B patient-derived skin fibroblasts carrying the RAB7^K126R^ mutations were analyzed by Western blotting using anti-RAB5, anti-Rab4, anti-Rab9, anti-CI-MPR, anti-TGN46, anti-TSG101 and anti-EAP30 antibodies. (**B**,**D**) Bands were quantified using ImageLab software (Bio-rad) and normalized against Vinculin and MAPK1. (**E**) Multivesicular bodies were immunostained using anti-CD63 antibody. (**F**) CMT2B fibroblasts were incubated in full medium (FM) and in the presence of 400 nM bafilomycin A_1_ (BAF) for 24h. Then, cells were incubated in the presence of DQ-Red BSA and (**G**) fluorescence intensity analyzed by ImageJ software. Values are the mean ± SE of three different and independent experiments. * *p* < 0.05; *** *p* < 0.0001. Scale bar = 10 µm.

Furthermore, we analyzed expression of early endocytic RABs. We found comparable levels of RAB5, a protein controlling early endosomal homotypic fusion and transport from plasma membrane to early endosomes [44,45], as well as of RAB4, a protein regulating different steps of endocytic recycling [46] (Figure 6A,B). Interestingly, we found that RAB7^K126R^ fibroblasts were characterized by a significant increase in RAB9 compared to control cells (Figure 6A,B). Then, we evaluated the expression of cation independent mannose-6-phopshate receptor (CI-MPR), a protein involved in transport between Golgi and endosomes, and TGN46, localized to the trans-Golgi network [47,48,49]. The expression of CI-MPR and of TGN46 did not change in CMT2B fibroblasts (Figure 6C,D).

A crucial role in endocytosis is played by the sorting events controlling correct cargo shipment to lysosomes. Endosomal sorting complex required for transport (ESCRT) proteins are crucial for sorting at the level of early endosomes and are essential for targeting signaling receptors to degradation [50]. Considering this, we have investigated the expression of tumor susceptibility gene 101 (TSG101) and of ELL (eleven-nineteen lysine-rich leukemia) associated protein 30 (EAP30), the homolog of yeast VPS22, and we found that they are significantly decreased and increased, respectively. These results indicated that differently by classical CMT2B mutation, RAB7^K126R^ altered EAP30 expression and, thus, lysosomal biogenesis [51].

Moreover, we analyzed through confocal microscopy the abundance of multivesicular bodies by immunostaining of CD63, and no alterations of number, size or intracellular distribution of these organelles were found (Figure 6E).

Finally, in order to understand whether the higher lysosomal activity observed in CMT2B mutated fibroblasts was sensitive to the effect of lysosomal inhibitors, we have evaluated by DQBSA assay the effects of bafilomycin A1 on CMT2B fibroblasts. We have observed a reduced dequenching of this dye, indicating inhibition of BSA digestion and, hence, block of lysosomal degradation.

Altogether, these results indicate that in CMT2B fibroblasts carrying RAB7^K126R^ mutation, the abundance of early endocytic markers is not affected, but the increase in EAP30 expression and the accumulation of lysosomes suggest that this mutation alters lysosomal biogenesis.

## 4. Discussion

Altogether our results clearly indicate impairment of autophagy and increased lysosomal activity caused by expression of the CMT2B-causing RAB7^K126R^ mutant protein.

In particular, we demonstrated that CMT2B fibroblasts carrying the RAB7^K126R^ mutation are characterized by a very limited colocalization of the mutated RAB7 protein with LC3B. The decrease in RAB7^K126R^ localization on autophagic vesicles compared to control could indicate a strong influence of this mutant protein on the autophagic process with functional consequences. Autophagy is a dynamic process being characterized by a balance between autophagosome formation and its turnover upon delivery to lysosomes. Therefore, the addition of bafilomycin A1 to inhibit the vacuolar H^+^-ATPase and, hence, lysosome-mediated degradation, in physiological conditions blocks LC3B degradation and increases LC3B staining. Indeed, using bafilomycin in complete growth media, determining the increase in LC3B puncta, reflects the basal level of autophagy. In previous work, we have demonstrated that in CMT2B fibroblasts carrying the RAB7^V162M^ mutation the number of LC3B-positive organelles was strongly reduced compared to control fibroblasts in all tested conditions indicating inhibition of the autophagic flux [34]. Surprisingly, we also found in fibroblasts carrying the RAB7^K126R^ mutation a reduction in LC3B puncta in full medium and after bafilomycin treatment, but we did not see this reduction under starved conditions. This raises the possibility that the RAB7^K126R^ mutant blocking basal autophagy prevents fusion of mature autophagosomes with lysosomes but does not influence autophagy initiation or autophagosome formation. Cytometric analysis confirms our hypothesis by showing that cells expressing CMT2B-causing RAB7^K126R^ mutant protein were characterized by a reduction in autophagosome/lysosome fusion rate compared to RAB7 wild-type transfected cells.

Inhibition of autophagic flux is observed also in HeLa cells expressing RAB7^K126R^ and RAB7^V162M^ mutants compared to RAB7^wt^. Interestingly, in HeLa cells, we did not see an increase in LC3B-puncta after starvation, but this could be due to the presence of three functional copies of RAB7 encoded by these cells which probably reduces the overall effect of RAB7 exogenous expression.

Cellular wastes are eliminated by autophagy, which is an essential process above all in post-mitotic cells, such as neurons, characterized by very high and efficient basal autophagic activity. Indeed, protein aggregates and consequent cell damage occur when this route is dysfunctional [52]. For these reasons impairment of autophagy is recognized as the main cause of neuronal degeneration both in the central and peripheral nervous system [52,53,54,55,56]. Moreover, axonal and dendrite degeneration is induced by autophagy alterations because these cells are particularly sensitive to protein aggregates accumulation [57,58,59,60]. Furthermore, autophagy induction is a mechanism used by cells to determine a block of axonal degeneration [61,62]. Albeit the fibroblasts are not the ideal cellular model to study peripheral neuropathy, the use of patient fibroblasts has been found very useful to analyze the possible presence of alterations of vesicular traffic as it has been done for other neuropathies [63,64,65,66]. As consequence, it should be considered that individuation of autophagy dysfunction in fibroblasts reflects impairment of mechanism in neurons that, being post-mitotic cells, are much more sensitive to the accumulation of undegraded material. Furthermore, it is essential to underline that a peripheral nerve fiber is mainly composed of neuronal axons, Schwann cells and fibroblasts, which are critical components of all nerve compartments, including endoneurium, perineurium and epineurium [67]. Here, dysfunctional autophagy linked to expression of CMT2B-causing RAB7 mutant protein could induce loss of its neuroprotective function, in the long-term, supporting establishment of the CMT2B disease.

In addition, we previously demonstrated that fibroblasts carrying the RAB7^V162M^ mutation are characterized by alterations in lipid metabolism and by the accumulation of lipid droplets (LDs) [35]. LDs are specialized organelles finalized to lipid storage [68] and selectively degraded by autophagy through a process termed lipophagy [69]. It is known that RAB7 is the main regulator of the interaction between LDs and degradative compartments essential for initiation of LD catabolism through autophagy [15]. Therefore, dysfunctional autophagy is accompanied by dysfunctional lipophagy with potentially dramatic consequences on lipid metabolism [35] and, hence, on the nervous system. Indeed, lipids are essential for several nervous key functions from synaptogenesis to impulse conduction and a number of CMT forms are characterized by alterations in lipid metabolism. In this manuscript, we found that fibroblasts carrying the RAB7^K126R^ mutation display an accumulation of LDs. Indeed, the dimension of LDs can change depending on nutrient conditions, with an increase in their size in the case of fatty acid abundance or a decrease in their dimensions during starvation, when fatty acids are used for energy production through β-oxidation [70]. Indeed, we observed a significant reduction in the size of LDs of CTR cells after starvation (Table 1). Notably, although accumulation and increased size of LDs are evident in CMT2B cell carrying the RAB7^K126R^ mutation compared to control cells, these effects are much less pronounced if compared to what happens in cells carrying the RAB7^V162M^ mutation both in terms of accumulation and size of LDs. Moreover, RAB7^K126R^ mutated fibroblasts are characterized by a stable size of LDs after starvation, while fibroblasts carrying the V162M mutation undergo an increase in LD size in the same condition. In addition, in cells carrying the RAB7^K126R^ mutations, most LDs are not surrounded by CD63 and LAMP-1 positive degradative compartments compared to control cells, indicating an impairment of the lipophagy mechanism as described for V162M [35]. The milder phenotype regarding LDs caused by the RAB7^K126R^ mutant protein could be one of the reasons that justify differences in the observed predominantly motor phenotype in patients.

In this contest, it is important to note that fibroblasts carrying the RAB7^V162M^ mutation are characterized by higher lysosomal degradative activity [33]. Indeed, it was demonstrated that this mutation is associated with an increase in Cathepsin D maturation, an increase in EGFR degradation and increased lysosomal activity although the autophagic flux is inhibited [33]. This is explained by the fact that, although endosomal and autophagosomal processes share many machinery components, they also are characterized by important differences and can be regulated in different manners [71,72].

We have previously demonstrated that a substantial difference between K126R and V162M mutants is represented by a strong inhibition of EGFR degradation in RAB7^K126R^ expressing cells [22]. Furthermore, we demonstrated that the strong increase in the total amount of EGFR in patient cells harboring the RAB7^K126R^ mutation was due to impairment of EGFR trafficking to late endosomes and lysosomes with consequent inhibition of its degradation and accumulation in early endosomes. For these reasons, we have here investigated, in fibroblasts carrying the RAB7^K126R^ mutation, lysosomal degradative activity by analyzing firstly lysosomal abundance and then lysosomal activity through Cathepsin D maturation and DQBSA assays. Although we have monitored the maturation of cathepsin D just as a marker of the functionality of the endocytic pathway, it was demonstrated that inhibition of autophagy can induce lysosomal membrane permeabilization, impairment of autophagic degradation, and, finally, a large induction in cathepsin D [73]. Moreover, the treatment of tumor-bearing mice with LCL521, a lysosomotropic inhibitor of acid ceramidase, activates cathepsin B and cathepsin D, resulting in interrupted autophagy and ER stress [74]. These literature data support our results relative to the increase in mature cathepsin D accompanied to an impairment of the autophagic flux in CMT2B.

Interestingly, we observed also in these cells an increase in lysosomal abundance and activity, but not as strong as the ones observed in cells harboring the RAB7^V162M^ mutation. Increased lysosomal activity is able to induce activation of feedback control mechanism determining decrease in ESCRT proteins [33]. Interestingly, our data are also supported by an increase in EAP30 protein, different to what is reported in CMT2B classical mutated fibroblasts [33]. We think that this can be explained by the fact that the moderate increase in lysosomal activity in CMT2B carrying the RAB7^K126R^ mutation is unable to induce the negative feedback control on the ESCRT system. This behavior is coherent with the intermediate molecular phenotype observed in relation to impaired autophagy and lipophagy in fibroblasts harboring RAB7^K126R^ with respect to RAB7^V162M^-mutated and healthy cells.

In light of these data, we speculated that the different predominantly motor patient phenotype in the case of RAB7^K126R^ mutation could lie in the observed differences in EGF receptor degradation and its relationship to the autophagy pathway. Indeed, it is known that beyond canonical functions EGFR has non-canonical roles such as regulation of autophagy and metabolism [75]. In particular, it was described that ligand-activated EGFR may have dual activity finalized to stimulate cell proliferation and to induce inhibition of autophagy [75] and this is coherent with faster growth of these cells compared to V162M mutated fibroblasts (data not shown). Moreover, it was recently demonstrated that autophagy inhibition alters EGFR trafficking resulting in its accumulation in early endosomes as we observed only in K126R fibroblasts [76]. Thus, we hypothesized the occurrence of a vicious circle in which EGFR accumulation determines impairment of autophagic route that in turn influences EGFR accumulation in early endosomes. Nevertheless, the increased amount of EGFR is probably able to compensate at minimum dysfunctional autophagy, inducing activation of proliferation and minimal basal metabolic functions justifying a slight autophagy/lipophagy impairment and degradative activity.

## 5. Conclusions

In conclusion, our data indicate that the CMT2B-causing RAB7^K126R^ mutation inhibits autophagic flux and lipophagy. Nevertheless, comparing cells harboring the RAB7^K126R^ mutation with cells with the classical RAB7^V162M^ mutation we found that these alterations are not so strong, probably due to the EGFR pathway compensation, albeit this not sufficient to prevent the occurrence of CMT2B symptoms. This information, therefore, suggests that the possibility that targeting autophagy may represent a potential approach to improve the conditions of patients with both CMT2B-causing *RAB7* mutations.

## Figures and Tables

**Table 1:** Lipid Droplet Characteristics Determined as Determined by Microscopy.

	Growth Condition	N° Lipid Droplets/Cell	Average Size (µ2)	Colocalization Rate vs. CD63	Colocalization Rate vs. LAMP-1
CTR	FM	118 ± 18	0,020 ± 0,007	0,993 ± 0,052	0,997 ± 0,0005
	ST	112 ± 3	0,0048 ± 0,009 **	0,495 ± 0,06 *	0,589 ± 0,05 **
K126R	FM	275 ± 49 *	0,022 ± 0,006	0,432 ± 0,1 *	0,344 ± 0,03 ***
	ST	332 ± 35 ***	0,027 ± 0,008 *	0,215 ± 0,01 *	0,285 ± 0,07 *
V162M	FM	395 ± 59 **	0,039 ± 0,001 *	0,274 ± 0,03 ***	0,355 ± 0,12 *
	ST	271 ± 57 *	0,058 ± 0,01 **	0,173 ± 0,1 **	0,18 ± 0,03 ***

* *p* < 0.05; ** *p* < 0.01; *** *p* < 0.0001.

## Data Availability

The data presented in this study are available in article.
